# Coastal change in Southeast Asia from geological to contemporary timescales: a review

**DOI:** 10.1017/cft.2026.10039

**Published:** 2026-06-16

**Authors:** Paul S. Kench

**Affiliations:** Coastal Marine Group, School of Science, The University of Waikato, Hamilton, New Zealand

**Keywords:** coastal change, sea-level rise, tectonics, coastal geomorphology, morphodynamics

## Abstract

Southeast Asia’s coasts are among the world’s most physically and ecologically sensitive environments, facing escalating threats from climate change, rising seas, extreme events and pervasive land subsidence. Understanding the magnitude, rate and trajectories of coastal transformation is critical for adaptation planning and hazard mitigation. This review synthesises current knowledge of coastal landform change across Southeast Asia (SEA), emphasising spatial and temporal variability driven by geological, climatic and anthropogenic boundary conditions. While globally sea levels are rising, tectonic deformation and vertical land motion dominate local trajectories, resulting in large spatial variability in relative sea-level rise (RSLR), offsetting SLR in uplifting areas and amplifying it in subsiding areas, where rates exceed 8 mm/yr, rivalling those of the Holocene marine transgression and surpassing global averages. The evolution of coastal landform types in response to boundary controls is examined. Paleo-reconstructions reveal that the Holocene transgression inundated ~2.3 million km^2^ of the Sunda Shelf, fundamentally resetting the land–sea interface of SEA countries. Sea-level fall from the mid-Holocene highstand drove extensive coastal deposition, generating >100,000 km^2^ of lowlands over the past 6,000 years, with progradation rates of 10^1^–10^2^ m/yr. These geologically young landscapes now face rapid, multidirectional change under contemporary forcing. Coastal change in SEA is complex, and rather than uniform erosion and inundation, the region exhibits a mosaic of responses, from stability to rapid progradation and island migration. Contemporary rates of change are comparable to those documented across the Holocene (10^2^ m/yr), but are responding to a broader suite of drivers, including tectonic deformation, variable RSLR, extreme events and intense human activity. Attribution remains elusive, constrained by sparse spatial coverage, uneven representation of landform types and limited temporal resolution of data. Addressing these gaps requires comprehensive, site-specific studies of both geological and contemporary dynamics across all coastal typologies, supported by high-resolution monitoring and integrated modelling. Such efforts will provide the empirical foundation needed for informed coastal management and adaptation strategies in one of the world’s most vulnerable regions.

## Impact statement

Coastal systems in Southeast Asia are home to hundreds of millions of people and some of the planet’s most productive ecosystems, yet they are among the most vulnerable to sea-level rise and climatic change. Rising seas, land subsidence and extreme weather events are reshaping coastlines, threatening livelihoods, infrastructure and biodiversity. This review provides the first comprehensive synthesis of how the coasts of Southeast Asia have evolved over thousands of years and how they are responding today to accelerating environmental pressures. By integrating geological records with modern observations, the study reveals that rates of contemporary coastal change are similar to those identified during the Holocene (last 10,000 years), the last period of substantial global sea-level rise. Importantly, the review demonstrates that coastal change is not uniform. While some areas are eroding and are subject to increasing inundation events, others experience rapid land expansion, uplift or island migration. These findings challenge simplistic narratives of inevitable coastal loss and highlight the need for locally tailored adaptation strategies. The implications extend beyond Southeast Asia. Understanding how tectonics, sea-level rise and human activity interact to shape coastlines offers critical insights for other coastal regions worldwide. The review also identifies major knowledge gaps and provides a direction for future research to improve the knowledge base of coastal change through site-specific studies of both paleo and modern dynamics across all coastal types, supported by high-resolution monitoring and integrated modelling. Ultimately, this work provides a scientific foundation for decision-makers, planners and communities to anticipate future risks and design resilient coastal systems. In doing so, it contributes to global efforts to safeguard vulnerable coastlines in an era of unprecedented environmental change.

## Introduction

Coastal communities across Southeast Asia (SEA) face mounting threats from climate change, including accelerating sea-level rise, intensifying storm surges and widespread land subsidence (Seah, 2022; Dong et al., 2024). These combined forces are reshaping shorelines, eroding natural defences and amplifying risks to livelihoods, infrastructure and ecosystems. Understanding and resolving how physical coasts are changing is critical, not only to safeguard vulnerable populations but also to inform sustainable adaptation strategies that can withstand the dynamic pressures of a rapidly evolving coastal environment.

The coastline of SEA extends more than 108,700 km in length and delimits the land-sea interface of 10 nations (Cambodia, Myanmar, Vietnam, Thailand, Malaysia, Brunei, Philippines, Singapore, Indonesia and Timor-Leste). Spanning the subtropics in the north (Vietnam) to tropical equatorial climates in the central and southern sectors, the environmental and geophysical setting of the region is complex and imparts a unique imprint on the diversity, morphological development and dynamics of the region’s coastal systems. Conspicuous coastal landforms include beach ridge plains, deltas, more than 25,000 islands, coral reefs, extensive mangrove systems, estuaries and rock coasts (Wong, 2005; Table 1).

**Table 1.** Summary of coastal attributes and dominant coastal landform types of countries in Southeast Asia Table 1. long description.

Country	Coastal length (km)	No. islands	No. coastal studies*	Dominant shoreline characteristics
Brunei	130	35	0	Sandy coast dominates western coastline
				Mangroves dominate the east and in estuaries
Cambodia	400	50	3	Mangrove-dominant coast. Pocket beaches bounded by rock headlands
Indonesia	81,000	17,500	10	Mangrove extensive on the east coast of Sumatra, east and south Kalimantan and southern Papua
				Rock coast dominates Sulawesi, the Lesser Sunda Islands and southern coasts of Sumatra and Java
				Coral reefs are widespread, including fringing, barrier and atolls
Malaysia	4,800	878	16	Mangrove systems dominate the west coast of Peninsula Malaysia, western Sarawak and west and east Sabah. Many infilled estuaries
				Sandy coast and beach ridge plains dominate the east coast of Peninsula Malaysia, NW and NE Sabah and eastern Sarawak
Myanmar	2,300	800	2	Ayeyarwady mega delta dominates central coastline
				Bold coast on the northwest
Philippines	13,000	7,640	7	Bold coast dominates exposed areas with pocket beaches
				Mangroves occupy sheltered areas
				Coral reef systems are extensive as fringing and barrier forms
Singapore	300	64	3	Anthropogenic modification of shoreline dominates; mangrove fringes northern coast
				Coral reefs present on offshore islands
Thailand	3,000	1,430	18	Mangroves dominate western coast of Peninsula
				Beach ridge plains, beaches and spits dominate west and eastern coastline of Gulf of Thailand, and SE coastline. Pocket beaches on SW Peninsula and islands
				Delta (Chao Phraya) dominates the head of the Gulf of Thailand
Timor-Leste	706	13	0	Rock/cliff coast in north with pocket beaches and fringing reefs
				Broad coastal plains and sedimentary shorelines in the south
Vietnam	3,800	4,000	43	Deltas dominate the north (Song Hong) and south (Mekong)
				Sand coasts (beaches, barriers), estuaries dominate the central coast

*Note*: Adapted from Wong (2005). *Data from Dong et al. (2024) of studies of coastal change in each country.

A distinctive feature of coastal landforms in the region is the role of ecological processes that moderate geomorphic development and change. The region hosts one-third of the Earth’s mangrove systems (47,000 km^2^), which modulate progradation of coastal lowlands in estuaries, deltas and lower-energy sedimentary shorelines. In addition, the region embraces the Coral Triangle recognised as a hotspot of coral biodiversity, species richness and abundance. The region supports 34% of the world’s tropical coral reefs across an area of 100,000 km^2^ (Hoeksema, 2007). These coral reef systems provide substrate and nutritional sources for coastal communities and coastal protection (Kench and Mann, 2017). These tropical bio-geomorphic systems are sensitive to variations in sea level.

SEA is also one of the most densely populated coastal regions in the world, supporting 500 million people, though there are extreme variations in population density between urban and rural coastal communities. The region has four low-lying coastal megacities (Manila, Jakarta, Ho Chi Minh City and Bangkok) where population densities reach ~20,000 people/km^2^. Such urban concentration places significant pressure on infrastructure and coastal ecosystems, leading to issues such as pollution, ecosystem degradation and heightened vulnerability to climate-related disasters (Tao et al., 2025). In contrast, rural coastal communities have much lower population densities and maintain a dependence on small-scale fisheries, aquaculture, mangrove forests and coral reefs for core economic activities. Seafood resources alone provide food security and income for over 130 million people in the Coral Triangle (Cruz-Trinidad et al., 2014). The coastal systems upon which communities dwell and depend also afford geomorphic services, such as natural coastal defences, reducing erosion and buffering villages from storms (Reguero et al., 2018). Consequently, the coastal communities in SEA remain highly dependent on healthy marine and coastal ecosystems, and their resilience is closely tied to the preservation and sustainable management of these natural environments.

In addition to a range of anthropogenic stresses on coastal systems (Culhane et al., 2024), climatic change and sea-level rise pose major threats to coastal communities throughout SEA (Oppenheimer et al., 2019). There is global consensus that coasts of SEA are most at risk to rising sea level, with the broader region host to 70% of the world’s coastal population (Nicholls, 2021; Seah, 2022). Sea levels across the region are expected to rise at rates faster than the global average (Fox-Kemper et al., 2021; Ng et al., 2024) and it is recognised that sea levels will continue to rise for several centuries beyond 2,100 and remain elevated for thousands of years (Oppenheimer et al., 2019; Fox-Kemper et al., 2021). Such large excursions in sea level will have profound effects on the physical coastlines of SEA. Coastal erosion, loss of land, migration of coastal landforms, submergence, increased flooding, inundation and salinisation are commonly cited impacts of sea-level rise and altered ocean wave regimes (Oppenheimer et al., 2019). While such transformations will exert stress on coastal ecosystems (Saintilan et al., 2023; Toimil, 2023), global impact assessments indicate annual economic impacts are on the scale of US$ trillions annually, in lost ecosystem services, disrupted and damaged economic services, in addition to the loss of habitability and displacement of millions of people (Islam and Khan, 2018; Monioudi et al., 2025).

Despite substantial research efforts to resolve global and regional rates of sea-level change, there has been comparatively little focus on how sustained increases in sea level will transform the physical substrate of coasts in SEA and the human and natural systems they sustain. The precise magnitude and rates of physical change in coastal landforms remain uncertain, and no robust projections exist for how coasts will physically transform over the next century, constraining adaptation responses (Ranasinghe, 2020; Toimil, 2023).

This study examines the state of knowledge of coastal landform dynamics in SEA. It explores the complex geophysical boundary controls on coastal formation, provides an account of coastal landform development in response to sea-level change since the end of the last glaciation and subsequent Holocene marine transgression, and examines state of knowledge of the rates and styles of contemporary coastal change.

## Boundary controls on coastal systems of Southeast Asia

### Geophysical boundary controls

**Figure 1.**
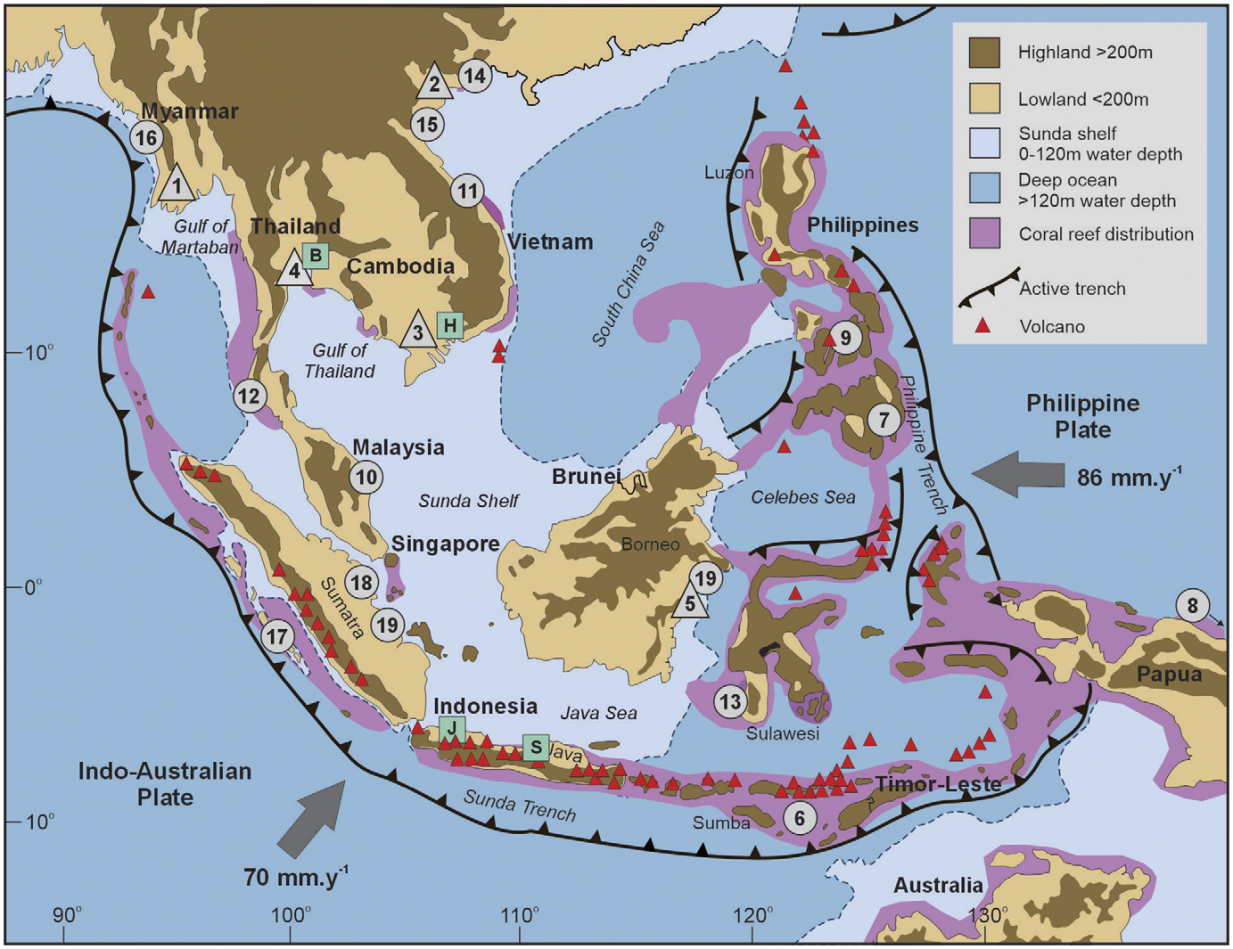
Location map of countries of Southeast Asia highlighting major oceanic plates, active plate margins and volcanic cones. Numbers refer to locations cited in text. Triangles denote deltas: 1-Ayeyarwady, 2-Song Hong, 3-Mekong, 4-Chao Praya, 5-Mahakan. Circles denote locations cited in text: 6- Lesser Sunda Islands, 7-Davao, 8-Huon Peninsula, 9-Cebu, 10-Terengganu, 11-Chan May, 12-Phuket, 13-Spermonde archipelago, 14-Beilun estuary, 15 Nghe An Province, 16 Rakhine state, 17-Mentawai Islands, 18-Riau Province, 19-Jambi Province, 20-Kalimantan Timur. Squares denote cities cited in text: S-Semarang, J-Jakarta, H-Ho Chi Minh City, B-Bangkok.

Active tectonic processes have established the broad physiographic coastal architecture and sediment transport pathways of SEA across geological timescales, which continue to impart critical controls on landscape dynamics. The land masses and coasts of SEA have formed at the boundary of active collision of three major plates and multiple micro-plates (Figure 1). The central land mass of SEA (Sundaland) comprises the southern extension of the Eurasian continental plate that terminates in Singapore and subsequently continues beneath the Straits of Malacca and the shallow Sunda shelf, forming Sumatra and northwest Borneo. These interior sections of SEA, including the Java Sea, shallow Sunda shelf and continental areas of Sundaland, are tectonically quiet (Hall, 2017).

In contrast to the relatively quiet tectonic central areas of Sundaland and the Sunda Shelf, the southern and eastern margins of SEA experience active tectonism through the collision of oceanic plates with Sundaland (Kessler and Jong, 2016). To the west and south, the Indo-Australia plate is moving northward from the Indian Ocean spreading axis at a rate of ~70 mm/yr and subducts beneath the Eurasian plate, forming the deep Sunda/Java trench. To the east, the Philippine Sea Plate is pushed westward by the Pacific plate at a faster rate of 86 mm/yr and subducts beneath the Eurasian plate, forming the Philippine Trench. These plate collisions produce a complex suite of volcanic arcs and subduction zones along the southern and eastern margins of the region. For example, an arc of volcanic and non-volcanic islands, related to the Sunda subduction system, links Sumatra with Papua. In addition, a complex arc system associated with the Philippine Trench connects northwest Papua, Sulawesi and the Philippines (Figure 1).

The coastal geomorphic imprint of subduction zones along the southern and eastern boundary of SEA is expressed as vertical land movement (VLM), resulting in uplift or subsidence at coastlines often hundreds of kilometres from trench axes (Zachariasen et al., 1999). While there remain significant geographic gaps in resolving such processes (Kessler and Jong, 2016), several studies do provide insights on the spatial variability and rate of VLM. Rates of long-term uplift over the Holocene are variable and include rates of 0.5–0.65 mm/yr at the Mentawai Islands, southern Sumatra (Zachariasen et al., 1999); 0.2–0.65 mm/yr at Sumba Island (De Gelder et al., 2023) in the Lesser Sunda Islands, SE Indonesia; 0.17–0.82 mm/yr in NW Luzon, Philippines (Maxwell et al., 2018); and 0.7–3.5 mm/yr at the Huon Peninsula, Papua New Guinea (Chappell et al., 1996). While these values reflect long-term rates of uplift, studies also highlight that uplift is commonly driven via episodic coseismic events. For example, the 2004 Sumatra-Andaman earthquake caused several metres of coseismic uplift in northern Sumatra (Meltzner et al., 2006). Similar observations have been reported in the Huon Peninsula, with individual coseismic uplift events ranging from ~0.1 m (Pandolfi et al., 1994) to 1–3 m (Chappell et al., 1996; Zachariasen et al., 1999). In the Mentawai Islands, Zachariasen et al. (1999) also noted that uplift is commonly preceded by notable subsidence of a similar magnitude, resulting in a near balanced tectonic signal.

Coastal subsidence also has a complex spatial pattern across the region which has multiple process drivers. Subsidence occurs through post-seismic viscoelastic deformation at greater distances from rupture zones. For example, the Andaman coast of Thailand has recorded persistent subsidence following the Sumatra-Andaman earthquake, as confirmed through subsiding coral reefs and coral growth responses (Simons et al., 2019). In addition to tectonic drivers, anthropogenic activities have exerted a major influence on subsidence, particularly in coastal cities where groundwater extraction has resulted in sediment compaction. For example, rates of subsidence reach 100 mm/yr at Semarang on the north coast of Java and 60 mm/yr in Jakarta (Bott et al., 2021), 21.7 mm/yr in Bangkok, and 16.2 mm/yr in Ho Chi Minh City in the Mekong Delta (Tay et al., 2022). Sediment compaction can also contribute to land subsidence in mangrove and peatland systems, such as the northeast coast of Borneo (Peng et al., 2024), and this compaction can also be amplified by anthropogenic impacts.

**Figure 2.**
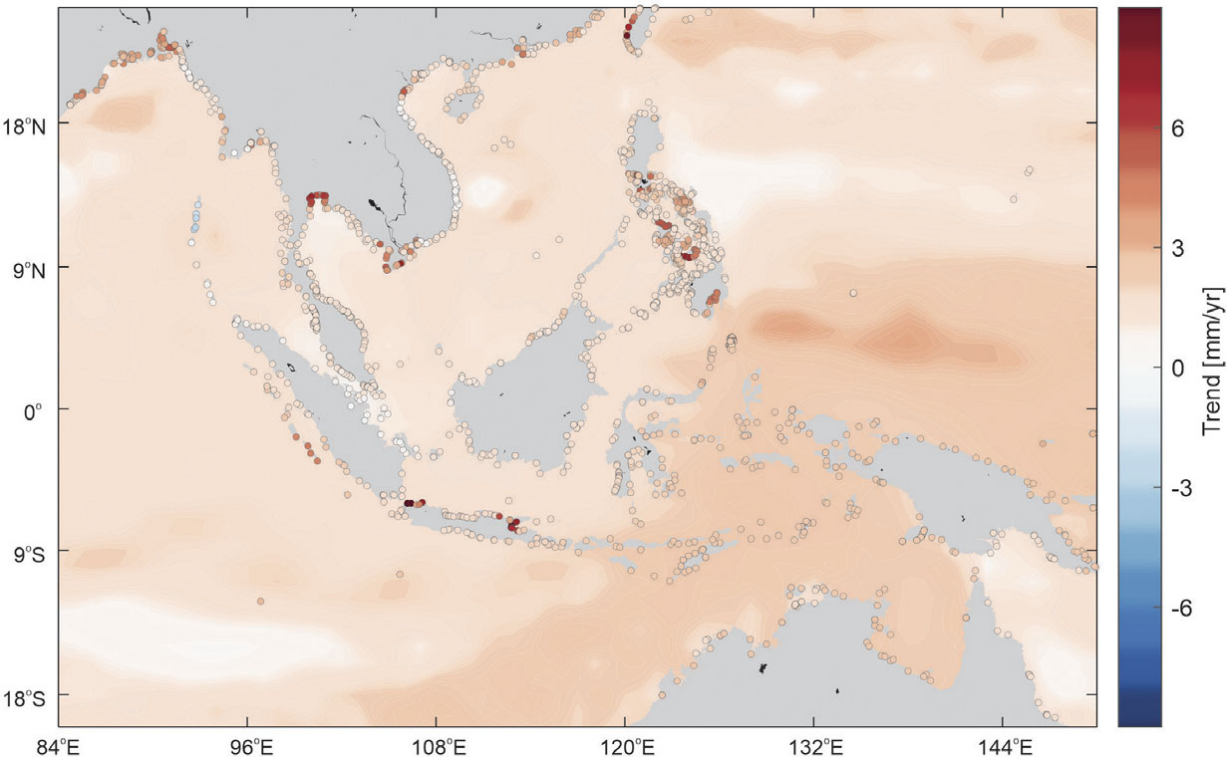
Trends in relative sea-level change (mm/yr) across the SEA region for the period 1900–2021 from the sea-level reconstruction (sum of sterodynamic effects of gravitation, rotation and deformation (GRD) related to present- day barystatic sea-level change, inverse barometer effects and glacial isostatic adjustment) produced by Dangendorf et al. (2024). Circles are coastal segments for which residual vertical land motion due to non-GIA processes was added based on a combination of GNSS, inSAR, ALT-TG and CSL-TG (Oelsmann et al., 2024; Dangendorf et al., 2026; Oelsmann et al., under review).

In summary, active tectonism has exerted a major influence on coastal landform development at locations proximal to the subduction zones at the southern and eastern limits of SEA. The spatial variability in rates of VLM is significant for current and future coastal trajectories, particularly in the context of relative sea-level change. Where uplift rates are greater than global sea-level rise, coastlines may experience relative emergence. Examples include large areas of Borneo where uplift has outpaced SLR since the late Pleistocene, causing relative sea-level fall and coastal emergence (Kessler and Jong, 2016). Coastal emergence is also recorded in the raised coral terrace and bold coasts of islands along eastern Indonesia and Sumatra (Meltzner et al., 2006; Maxwell et al., 2018; De Gelder et al., 2023). In other areas where uplift equals rates of sea-level change, there would be no change in relative sea level. Lastly, subsidence compounds the SLR signal, resulting in rates of relative sea-level rise (RSLR) much higher than the global average. Collectively, these differential tectonic signatures create a spatially variable RSLR pattern across the region (Figure 2). It is also important to note that the central mainland and Sunda Shelf are less influenced by tectonic processes, and at these locations eustatic SLR is a more prominent driver of coastal change. Consequently, the mixed RSLR signatures make SEA an excellent natural laboratory to examine coastal response to relative sea-level change.

### Sea level change

Long-term changes in relative sea level exert a major influence on the formation of coastal landforms, particularly in those areas not impacted by strong and ongoing tectonic signals. The pattern of Holocene sea-level change in SEA has been influenced by a combination of eustatic, isostatic and local factors (Mann et al., 2019). Located far from sites of glaciation, deglacial isostatic processes have had minimal influence on the Holocene RSL in SEA. Rather, hydro-isostatic processes, continental levering and equatorial siphoning (the redistribution of water from the tropics to higher latitudes following collapse of near-field peripheral forebulges) have controlled glacial isostatic adjustment in the equatorial regions (Lambeck et al., 2014).

**Figure 3.**
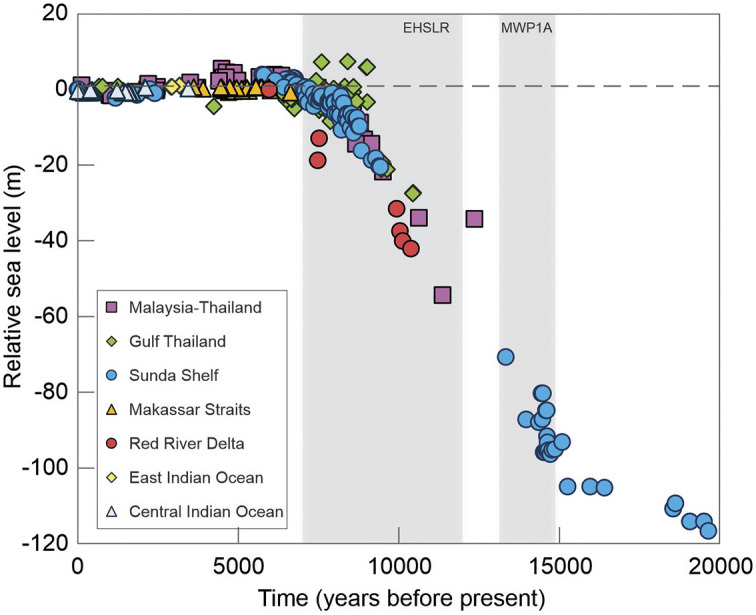
Pattern of Holocene sea-level change in Southeast Asia. Age elevation plot of standardised Holocene RSL indicators in Southeast Asia over the past 20,000 years compiled from the SEAMIS database after Mann et al. (2019) and data from Shaw et al. (2023), Kench et al. (2020) and Bender et al. (2020). Vertical grey shaded areas denote the periods of rapid sea-level rise: Meltwater Pulse 1A (MWP1A) and Early Holocene Sea-Level Rise (EHSLR).

The timing and rates of Holocene sea-level change in SEA have been defined from proxy records that include estuarine mangrove sediment sequences (Horton et al., 2005; Chua et al., 2021), coral reefs and microatolls (Parham et al., 2014; Mann et al., 2019; Bender et al., 2020; Kench et al., 2020) and other preserved sea-level indicators such as oyster beds and beachrock (Tjia, 1996). A synthesis of these datasets establishes a broad regional pattern of sea-level behaviour, which depicts a rapid increase in sea level following the late Pleistocene deglaciation (beginning 18,000 years ago) and first reaching near present sea level approximately 6,000–7,000 yr BP (Figure 3). Evidence from the Malaysian peninsula and Sunda shelf indicates the pattern of SLR was not uniform. For example, during meltwater pulse 1A (14.7–13.5 kyr BP, Figure 3), the rate of SLR was up to 15.4 ± 8.2 mm/yr (Shaw et al., 2023). Sea levels in the region rose above present levels 7 to 4 kyr BP, though regional differences have been found in the timing and elevation of this mid-Holocene highstand (MHHS; Mann et al., 2019; Zhang et al., 2025). On the Sunda shelf, a highstand is interpreted 6 to 4 kyr BP, which reached between +2 and +5 m above present level (Mann et al., 2019; Chua et al., 2021; Shaw et al., 2023). In the Java Sea, evidence for a highstand occurred between 7 and 5.5 kyr BP, ranging from +1.2 to +1.8 m (Meltzner et al., 2017), while evidence from the broader region suggests the highstand occurred later in the Holocene (3.5–2.0 kyr BP) at lower amplitude (+0.5 to +1.0 m, Mann et al., 2019). Following the MHHS, data show a subsequent fall in RSL in the late Holocene to near present levels as a consequence of equatorial siphoning, though proxy evidence has been unable to define the precise onset and synchronicity of this RSL fall. Sea level is thought to have been relatively stable for the past 800 years (Tan et al., 2024) until the recent acceleration.

### Climate and ocean processes

**Figure 4.**
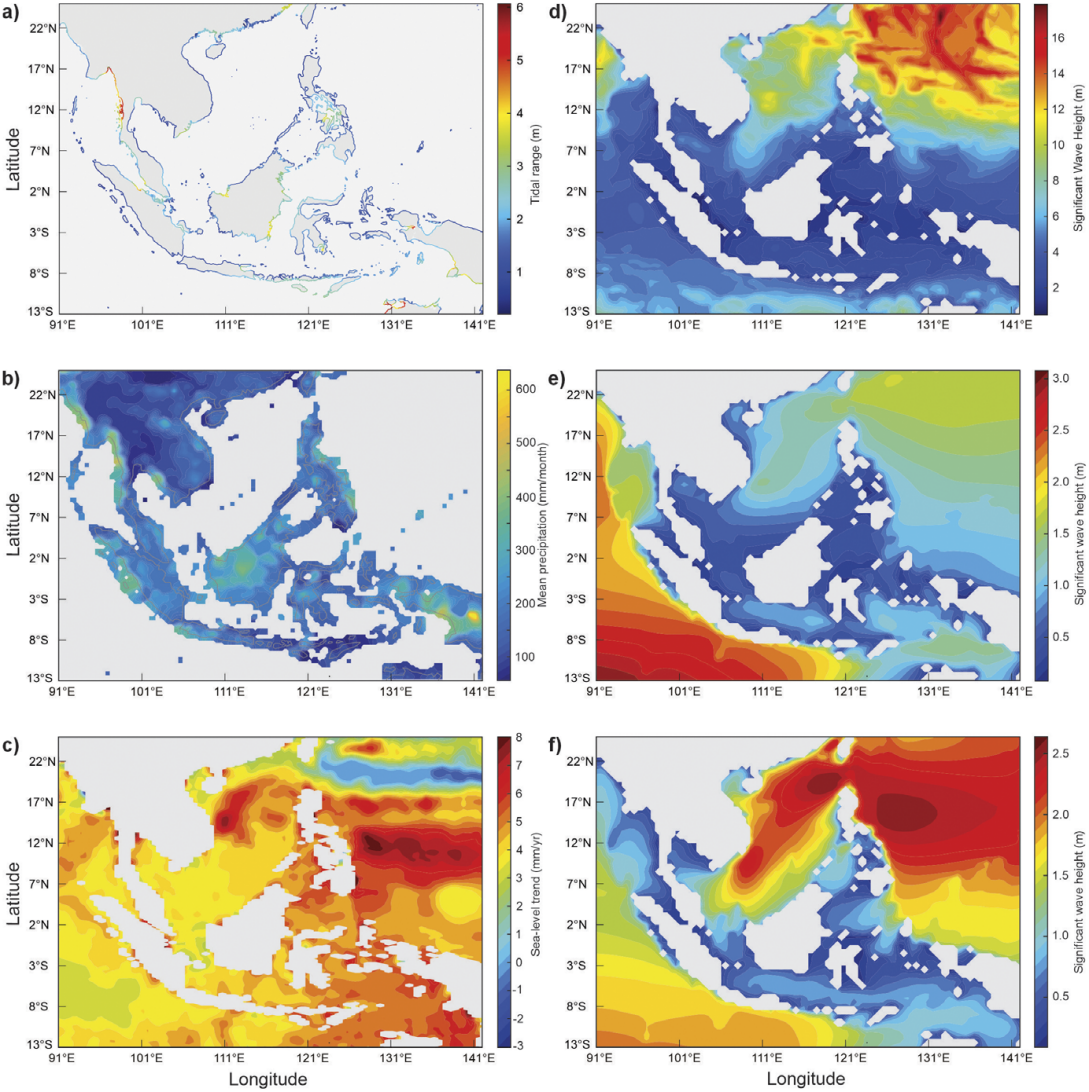
Summary of key coastal process boundary controls on coastal landforms of Southeast Asia. (a) Mean tidal range from ECMWF (1985–2014) Copernicus Climate Change Service (2022). (b) Mean monthly precipitation 1993–2014, *Source:* https://opendata.dwd.de/climate_environment/GPCC/PDF/GPCC_intro_products_v2018.pdf. (c) Mean sea-level trend 1993–2015, *Source:* ESA Sea Level CCI project team (2017), https://data.ceda.ac.uk/neodc/esacci/sea_level/data/IND/v2.0/MSLTR. (d) Mean maximum significant wave height (Hs) 1994–2023. (e) Mean Significant wave height (Hs) southwest monsoon 1994–2023. (f) Mean significant wave height (Hs) northeast monsoon 1994–2023. Wave data sourced from ECMWF ERA5, https://cds.climate.copernicus.eu/datasets/reanalysis-era5-single-levels?tab=overview.

In addition to geophysical and long-term sea-level change, coastal landforms in the region have evolved and continue to dynamically adjust in response to the contemporary coastal process regime, which exhibits marked spatial gradients in tidal range, wave energy, storm frequency and intensity, and varied patterns and rates of sea-level rise (Figure 4). The process regime is regulated by the broad-scale climate processes and oceanographic factors. Of note, SEA acts as a critical connection between the Pacific and Indian Oceans, where the Indonesian Throughflow transports warm, low-salinity waters from the western Pacific through the Indonesian archipelago to the Indian Ocean (Gordon et al., 2019). Ocean processes are also influenced by broad-scale climate modes of ENSO (Cheng et al., 2016) and the IOD (Han et al., 2019).

The region is primarily influenced by micro- to upper mesotidal conditions, which on low-gradient coasts create extensive intertidal zones and modulate the level of wave attack at shorelines. Macrotidal conditions are only found in isolated regions such as the southeastern shoreline of Myanmar (Figure 4a). The region is also subject to high rates of rainfall, which combined with steep relief and warm temperatures results in the highest denudation rates on earth (Syvitski et al., 2022). The significance for coastal landforms is the delivery of vast quantities of sediment to coastal systems, though such inputs are spatially concentrated.

Wave climate across SEA is strongly modulated by large monsoonal shifts in wind direction. During the southwest (May to September) monsoon, persistent southwesterly winds generate larger, longer-period ocean swells resulting in highest waves (mean Hs values >2.6 m) along the southwest margins and southeastern islands of Indonesia, the coast of Myanmar and west coast of southern Thailand (Figure 4e). During the northeast monsoon (November to March) there is a marked increase in wave energy along the eastern margin of the Philippines, Vietnam, Thailand and Malaysia (mean Hs values >2 m, Figure 4f). Wave heights in the central Sunda Shelf region are generally low to moderate (Hs <1 m) due to fetch limited constraints, though there are subtle monsoonal variations in Hs that impact shorelines. Maximum wave heights (Hs > 10 m) occur in the northeast of the region (Figure 4d) associated with the generation of tropical cyclones that form in the western Pacific Ocean and move across the northern Philippines towards Vietnam, and occasionally the coast of Thailand (Tran et al., 2022).

Sea-level change over the SEA region shows large regional variability on temporal scales from seasons to decades (Luu and Tkalich, 2023). Wind stress associated with oscillating monsoon seasons forces seasonal variability in sea level in the region by up to ±0.25 m. Positive sea-level anomalies occur in the southern and western areas of SEA during the NE monsoon, particularly in the Gulf of Thailand (~+0.25 m). These trends typically reverse during the southwest monsoon (Luu and Tkalich, 2023). At interannual scales, ENSO and IOD variability force variations in sea level between +0.3 m (La Nina) and −0.28 m (+ve IOD) (Mohan and Vethamony, 2018).

Over longer timeframes, sea level across the region has risen at an average rate of 4.4 mm/yr (1993–2021) and exceeded 6 mm/yr in the east (Figure 4c), which are higher rates than the global average. Significantly, regional differences in rates of RSLR are compounded by the influence of tectonic and anthropogenic processes (Figure 2). Due to these compounding factors, high rates of sea-level change are expected to continue. For example, projections at Cebu, Philippines indicate that by 2,150 sea level will rise by up to 3.05 m under higher sea-level scenarios (SSP5-8.5), at rates greater than 25 mm/yr (Ng et al., 2024).

## Coastal system response to Holocene sea-level change in Southeast Asia

**Figure 5.**
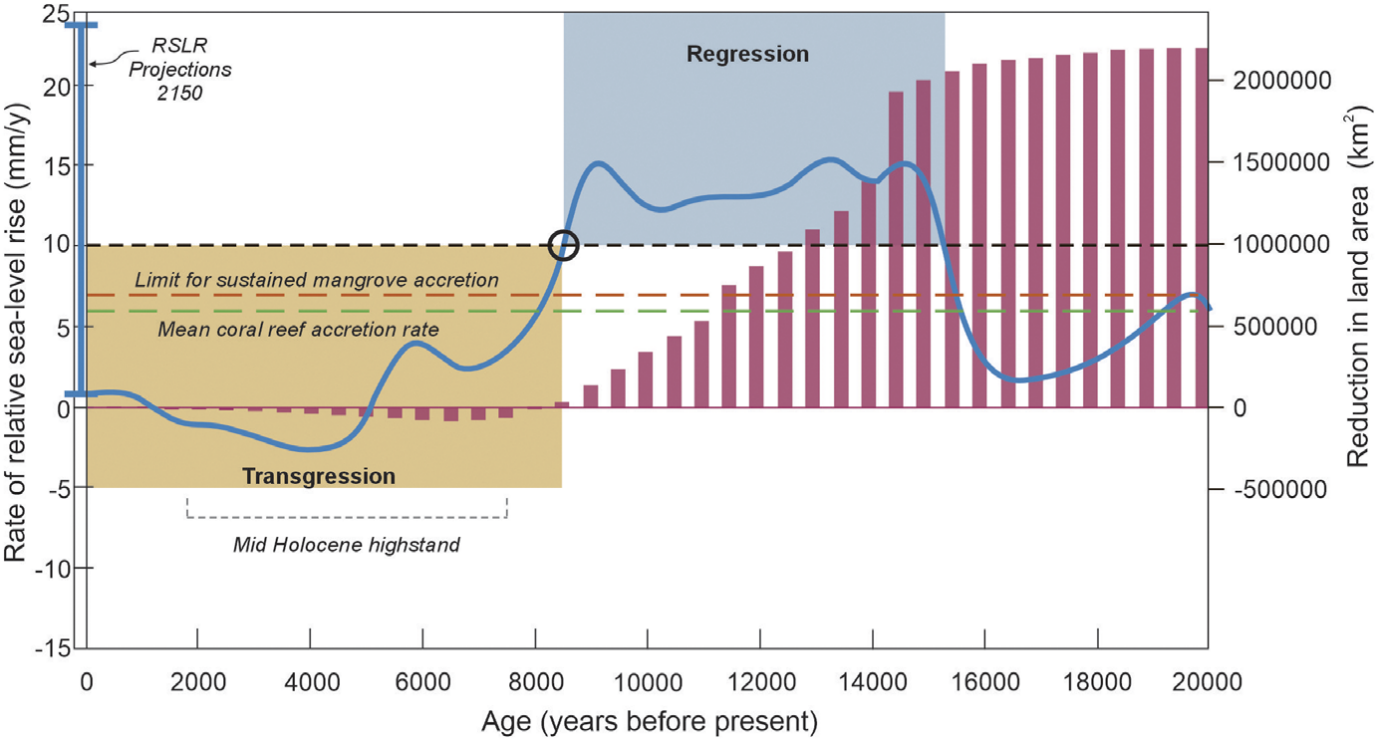
Rates of relative sea-level and coastal land area change on the Sunda Shelf, Southeast Asia during the Holocene marine transgression. Blue line denotes changes in the rate of relative sea-level rise based on geological reconstruction of the pattern of Holocene sea-level rise (Figure 2). Red bars depict the reduction in land area as the Sunda Shelf was flooded during the marine transgression (after Shaw et al., 2023). Also note the reduction in land area beyond present during the mid-Holocene highstand. Dashed orange line represents the upper limit of RSLR beyond which mangrove systems are unable to initiate sustained accretion following Saintilan et al. (2020) and (2023). Green dashed line represents regional rates of mean coral reef accretion from Spermonde archipelago after Hynes et al. (2025). Blue vertical line encompasses the range of projected rates of RSLR for SEA over the next century spanning both the SSP1-2.5 and SSP5-8.5 scenarios after Ng et al. (2024). Blue shaded box represents the period in the early Holocene when coastal trajectories were dominated by regression at rates of RSLR above 10 mm/yr. Beige box represents the period in the mid to late Holocene of active coastal aggradation and progradation (transgression) when rates of RSLR were below 10 mm/yr. Black dashed line denotes the transition from regressive to transgressive coastal responses around the RSLR rate of 10 mm/yr.

**Figure 6.**
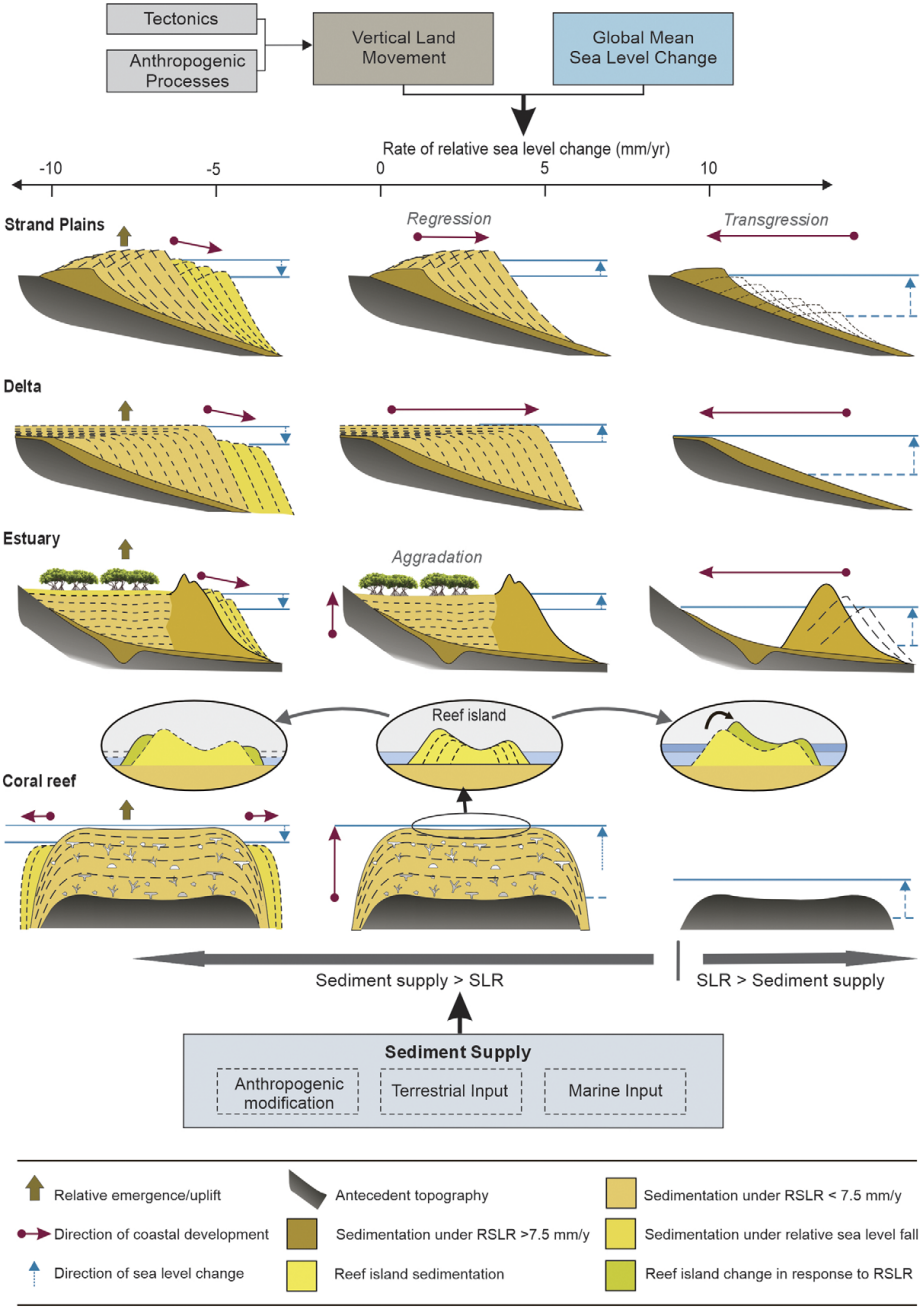
Schematic model of coastal behaviour in response to changes in relative sea-level rise and sediment supply. Five coastal typologies are presented: strand plains, estuaries, deltas, coral reefs and reef islands, with dominant coastal deposition and dynamics illustrated in response to both differences in RSLR and sediment availability.

Holocene sea-level change has governed the gross configuration of the coastline of SEA, and the period for landform development (Figures 5 and 6). First, rapid increases in sea level between 15,000 and 9,000 years ago at rates up to 15.4 ± 8.2 mm/yr promoted large excursions of sea level across the shallow regions of the Sunda Shelf. While coastal system response to marine transgressions is commonly considered in terms of vertical changes in sea level, less well recognised is the significant horizontal translation of coastal systems that may also compromise the ability of physical systems to adapt. At the height of the last glaciation, the land area of the region was much larger, with the majority of the shallow Sunda Shelf exposed and forming a land bridge connecting the islands in the region (Figure 1). Due to the shallow nature of the Sunda Shelf, mean rates of shoreline translation of ~57 m/yr occurred during the last deglacial transition, which increased to ~335 m/yr during Meltwater Pulse 1A, the period of rapid increase in sea level (Figure 5), before slowing to ~15 m/yr for the period after 10,000 years ago (Figures 3 and 5; Shaw et al., 2023). The land area of SEA reduced by approximately 2.3 million km^2^ as a result of the marine transgression (Figure 5). Periods of fastest rates of RSLR are likely to have compromised the ability of biogeomorphic systems (mangroves, estuaries and coral reefs) to adapt and keep pace with sea-level change (Figure 5). Specifically, RSLR greater than 7.1 mm/yr, which occurred for a 7,000 year period (8,000–15,000 yBP, Figure 4), is known to be beyond the likely capacity of mangrove and coral systems to initiate and keep pace with sea level (Saintilan et al., 2020, 2023; Hynes et al., 2025). Second, after 8,000 years ago the rate of sea-level rise declined to within the known range of adaptive capability of coastal systems to physically adjust and keep pace with RSLR (Figures 5 and 6). Consequently, the past 8,000 years has been characterised by active landform accumulation and progradation. Third, embedded in this latter period of reduced rates of SLR was a prominent sea-level highstand (~7,000–4,000 years ago) that varied regionally in elevation (0.5–5.0 m), and that occupied an additional 88,000 km^2^ of coastal area. Coastal landform development at these higher land-sea interfaces was constrained to a 1,500–4,000 year period. Fourth, over the last few thousand years these highstand deposits became emergent as sea level fell to near present by 1,000 years ago. Consequently, future increases in sea level will initially reoccupy these currently emergent paleo-coastal deposits that are 2,000–5,000 in age.

The depositional history of coastal landforms across SEA varies between landform types reflecting differences in antecedent topography, sediment supply, process regime and importance of ecological processes (Figure 6). The interaction of these factors imparts unique chronological and morphological properties on coastal landforms which are examined in greater depth for key coastal typologies.

### Beach ridge/strand plain development

Extensive beach ridge plains, ranging up to 16 km in width, formed in the incised valleys between headlands, as sea level invaded the coastline transporting sediments onshore and longshore. Studies from the Gulf of Thailand indicate establishment of landward beach ridges as early as 7–9 Ka (Leknettip et al., 2023; Polwichai et al., 2023), followed by active progradation (Figure 6). Notably, several studies highlight an increase in elevation of beach ridges to the peak of the mid-Holocene Highstand and a subsequent decrease in elevation that is correlated with the regional fall in sea level to approximately 1,500 years ago (Brill et al., 2015; Polwichai et al., 2023). A similar pattern of development is evident along the eastern coastline of Terenggannu, Peninsula Malaysia (Mallinson et al., 2014) and Chan May, central coast of Vietnam (Gouramanis et al., 2020), though the onset of deposition at these sites started later (~6,800 yBP) than sites in Thailand. Beach ridge plain development over the last few thousand years has also been a conspicuous feature of the recent active margins of delta systems (Ta et al., 2005; Giosan et al., 2018).

### Formation of deltaic estuarine plains

The formation of deltaic and estuarine plains, as a consequence of the Holocene marine transgression, created the largest expanses of coastal land in SEA. The four mega deltas alone account for 120,000–140,000 km^2^ of coastal lowlands, which were deposited over the past 8,000–6,000 years, with progradational sequences that extend from 60 km and 80 km in the Mahakan and Ayeyarwady deltas, respectively (Storms et al., 2005; Giosan et al., 2018), to 250 km in the Mekong delta (Xue et al., 2010) (Figure 1). Such extensive accumulation of sediment reflects the high sediment loads delivered to the coast, resulting in rapid seaward progradation (Figure 6).

Although the specific chronology of formation differs regionally, delta systems have followed a similar four-phase pattern of formation (Tanabe et al., 2003; Ta et al., 2005; Giosan et al., 2018). First, by 8,000 yBP the marine transgression had inundated incised valleys and embayments, delimiting the landward extent of Holocene coastal deposits (often hundreds of kilometres from the current shoreline), and transgressive estuarine muds had been deposited (Figure 6). Second, mangrove systems developed and expanded as rates of SLR decelerated toward the MHHS, driving rapid aggradation between 8,000 and 6,000 yBP that filled valley basins. Third, rapid progradation 6,000–3,000 years ago, at rates up to 40 km/ky, during the relative fall in sea level to present levels, (Ta et al., 2005). In places, relative sea-level fall created emergent delta surfaces, such as in the Song Hong delta (Tanabe et al., 2003). This phase of development has also been identified as critical in increasing the available land for rice cultivation in the region by 95,000 km^2^ (Ma et al., 2020). Fourth, continued progradation but at slower rates over the past 3,000 years.

A broadly similar pattern of coastal development is reported for estuarine systems in the region. Deposition of transgressive estuarine muds in incised valleys occurred from 10 to 8,000 years ago as sea level invaded the coast. Widespread mangrove forest development occurred around 8.5–7.5 ka in SEA as the rate of sea-level rise declined below 7 mm/yr (Saintilan et al., 2020), and mangroves associated with large rivers were able to maintain their intertidal position by trapping sediment and accumulating root mass, contributing to rapid estuarine infill 6–4.5 ka (Figure 6). This period of mangrove expansion was similar to the ‘big swamp’ phase reported in northern Australia (Woodroffe, 2000). As estuarine deposits prograded seaward, landward sectors of the estuarine plains experienced relative emergence due to sea-level fall and transition to fluvial dominance. Consequently, mangroves were replaced by peat swamps at many locations (e.g., northeast coast of Borneo, West coast of Malaysia and east coast of Sumatra; Nguyen et al., 2024).

### Coral reefs and reef island formation

Despite the region being host to the largest area of, and most biodiverse, coral systems on earth and containing a diversity of different reef morphologies, there is curiously little understanding of reef development in response to the Holocene marine transgression and how it influenced associated coastal landform trajectories. Along the southern and eastern tectonically active boundaries of the SEA region, flights of uplifted coral terraces of Holocene age are testament to active and persistent reef growth during the Holocene (Maxwell et al., 2018; Pedoja et al., 2018). Such terraces likely backstepped along the volcanic rock substrate during the marine transgression.

In tectonically passive settings, evidence indicates that coral reefs established as rising sea levels inundated Pleistocene reef substrates (e.g., the South China Sea and Sulawesi) and unconsolidated mud accumulations (e.g., Phuket and Singapore). The timing of reef initiation varies regionally, occurring at ~8.2–7.8 ka in the South China Sea (Ma et al., 2021; Zhao et al., 2022), ~7.5 ka in Singapore (Chan et al., 2025), ~7.2 ka in the Spermonde Archipelago (Hynes et al., 2024) and ~6–5 ka in Phuket, Thailand (Tudhope and Scoffin, 1994). Notably, these initiation phases coincide with a deceleration in sea-level rise to below ~5 mm/yr (Figures 3 and 6). Following initiation, reefs experienced rapid vertical accretion (4–8 mm/yr) between ~8 and 6 ka, enabling them to keep pace with, or catch up to, sea level during the mid-Holocene highstand. These rates are consistent with global estimates of reef growth (Hynes et al., 2024). After the highstand, reef growth rates declined markedly (to ~0–1.5 mm/yr) as vertical growth potential was reached, with some reefs becoming emergent in response to late Holocene sea-level fall. During this period, lateral progradation became the dominant mode of reef development (Hynes et al., 2025). Coral reef islands that are found throughout the reef systems of SEA are some of the youngest depositional landforms, and their formation was regionally controlled not only by sea level but also by the timing at which reef platforms approached their growth limit and provided the basement for island accumulation (Kench and Mann, 2017). Such basement can include primary reef growth or infill of shallow lagoons by sediment from the surrounding reef. Studies of island formation are scarce across the region. Recent evidence from the Spermonde archipelago suggests islands began forming ~3,800 years ago, after the MHHS and after sea level had fallen to present levels (Kappelmann et al., 2024). They further note that the generation of carbonate sediment for island building was modulated by sea level and monsoon behaviour and that islands have incrementally expanded over the past 2,000 years (Kappelmann et al., 2024).

## Contemporary coastal change in Southeast Asia

While coastal landforms exhibited large lateral displacement across the shallow Sunda Shelf during the HMT, followed by progradation of extensive depositional sequences over the past 8,000 years, recent studies based on remote sensing analysis have identified coastal transformations at comparable rates across SEA at much shorter decadal timescales. Analysis of the coasts of mainland SEA (excluding the Indonesian archipelago, the Philippines and offshore islands) found that across a 15-year timeframe (2000–2015) 18.5% of natural soft coasts had prograded (at a mean rate of 20.4 m/yr), while 11.3% have retreated (mean rate of −15.4 m/yr), with a net gain of natural coast of 534.3 km^2^ (Song et al., 2020; Dong et al., 2024). Approximately 70% of coasts remained stable across this timeframe. The study also highlighted a 29% increase (to a total of 21%) in artificial modification of coastlines in mainland SEA due to development and population growth (Song et al., 2020; Shaw et al., 2023).

The magnitude, rates and styles of coastal response differ between coastal landform types and such changes are spatially modulated by regional gradients in boundary controls and intensities of anthropogenic impact. Hotspots of coastal expansion are concentrated in Vietnam and Myanmar, which account for 44.4% and 25.65% of total coastal progradation in mainland SEA (Song et al., 2020). In Vietnam, the highest rates of coastal progradation were found in the Beilun River estuary (26.7 m/yr), the Song Hong Delta–Nghe An Province (22.5 m/yr), the central coast (5.0 m/yr) and the Mekong river delta (11.0 m/yr). Higher rates of progradation have been observed on the Myanmar coast along the north Rakhine state (67.9 m/yr) and between the Gulf of Martaban and Ayeyarwady delta (54.5 m/yr), corresponding to increased coastal areas of 581.9 and 338.8 km^2^, respectively, over a 15-year timeframe. Such rates of progradation are comparable to those identified in the Holocene and reflect an abundant sediment supply that is being actively deposited within available accommodation space. Cambodia, Malaysia and Thailand each had less than 15% of coastlines actively prograding at mean rates of 22.1, 15.0 and 8.1 m/yr, respectively.

Highest rates of erosion were also found in Vietnam (32% of erosion in mainland SEA), Myanmar (30.4%) and Thailand (24.8%) with mean rates of −6.7, −39.3 and −7.3 m/yr. Coastal erosion in Malaysia and Cambodia amounts to less than 10% of the SEA mainland total. High rates of shoreline retreat are also found on the northern Bay of Bangkok, and southern west coast of the Gulf of Thailand, which had mean rates of coastal retreat of −10.0 and −7.2 m/yr, respectively.

It has also been observed that maximum rates of accretion and erosion can be found at adjacent coastal sites. For example, in Myanmar, rates of coastal change between the Gulf of Martaban to Ayeyarwady delta range from 640.9 to −807.9 m/yr, while along the northern Rakhine coast rates vary from 593.6 to −269.7 m/yr (Song et al., 2020). Furthermore, a study of coastal change along the entire coast of Vietnam, over a 35-year period, found that 46% of the coastline was stable while approximately 27% had eroded and 27% had accreted. Notably, most substantive changes occurred around the low-lying deltas (Mekong and Song Hong Rivers) with maximum rates of change ranging from +47 to −28 m/yr (Lappe et al., 2022). Similarly high rates of erosion and accretion have been reported in deltaic systems of Indonesia (Solihuddin et al., 2021), and the northern coastline of the Gulf of Thailand over the past 75 years (Sok et al., 2022). Persistent erosion (−9 m/yr) at the Chao Phraya delta has been documented, with causes attributed to relative increase in sea-level and subsidence through groundwater extraction (Bidorn et al., 2021). Indeed, subsidence has been identified as a significant factor in enhancing relative SLR, impacting deltaic systems across the region (Nicholls, 2021). The spatial variability in trajectories of the soft sedimentary shorelines of SEA is also apparent in global assessments (Luijendijk et al., 2018; Vousdoukas et al., 2020), which provide indicative trends in physical change of similar rates and magnitudes reported here.

A key factor in the large differences in contemporary coastal change in mainland SEA is the relative importance of natural sediment supply and anthropogenic modification of the sediment system. Large progradation signatures in the Ayerwardy and Song Hong delta systems reflect high natural sediment inputs, and even enhanced inputs through actions such as deforestation (Van Cu et al., 2018; Latrubesse et al., 2021) as well as localised reclamation. However, areas of distinct erosion such as parts of the Mekong delta reflect a significant anthropogenically driven reduction in sediment supply through damming of rivers and canalisation of lower reaches of the delta surface (Tamura et al., 2020; Bussi et al., 2021).

In contrast to mainland SEA, examination of the change in islands of SEA (>9,000) shows a different range of morphological trajectories including expansion, contraction as well as positional movement and migration. More than 75% of the islands of SEA are less than 1 km^2^ while only 20 islands are larger than 10,000 km^2^. Examination of change over the past 25 years shows that 31% of island shorelines (a length of ~45,900 km) had changed position, with shoreline erosion and accretion occurring along 16% and 14.9% of island shorelines, respectively. Aggregated at the island scale, such changes resulted in 12.1% of islands (1,101) contracting in area and 10.4% (946) expanding in area, with a total loss of land area across all islands of 251 km^2^ (Zhang et al., 2021). Sites of greatest of expansion were located on the east coast of Sumatra (Riau and Jambi province), either side of the Singapore Straits, Jawa Barat in NW Java and north and southeast Sulawesi. Sites of greatest erosion were also located on the eastern coast of Sumatra and NW Java, as well as Kalimantan Timur and Papua Province. More recently Sengupta et al. (2025) examined shoreline dynamics of small coral reef islands in the Spermonde Archipelago and found island expansion was the dominant geomorphic adjustment in recent decades. Collectively, these studies highlight that unlike the mainland, islands exhibit different coastal responses, including positional movement, changes in island shape and shoreline length, and many islands are expanding in area (Zhang et al., 2021), as also found in tropical coral reef island settings throughout the Indo-Pacific (Kench et al., 2024). It is also noteworthy that in other small reef island settings studies have noted that island margins have the capacity to increase in elevation through overwash processes (Tuck et al., 2019).

## Discussion

Low-lying coastal landforms provide the foundation for human settlement, livelihoods and well-being for coastal communities across SEA. As demonstrated in this review, the coasts of SEA are dynamic landforms that are in continual adjustment to changes in environmental boundary conditions across geological to contemporary timescales. To date, aggregate global assessments of coastal change assume coasts will primarily erode and become submerged with increasing sea levels (Oppenheimer et al., 2019; Vousdoukas et al., 2020; Dong et al., 2024). Such assertions misrepresent the spectrum of coastal transformations that are occurring throughout the region (Woodroffe et al., 2025).

Paleo-reconstructions demonstrate that coastal systems in SEA have evolved, and undergone substantive transformation, in response to a spatially complex set of geological, climatic and anthropogenic processes. In particular, the Holocene marine transgression forced macro-scale landscape transformation through inundation of ~2.3 million km^2^ of land on the Sunda shelf, resetting the contemporary land-sea interface of SEA countries (Figure 5). Following the mid-Holocene highstand, a major phase of coastal deposition and emergence produced >100,000 km^2^ of habitable coastal lowland, with progradation rates ranging from 10^1^ to 10^2^ m/yr, depending on coastal type. Of note, these geologically young, and emergent, coastal landforms are undergoing active transformation in response to changes in environmental boundary conditions. Significantly, available evidence of coastal change spanning recent decades indicates the contemporary coastlines are changing and undergoing a broader set of system responses. Large sectors of coast have remained stable, while substantive sectors, particularly along the margins of the major delta systems, are actively accreting and eroding at rates comparable to those of the mid-Holocene (10^2^ m/yr). Similarly, coral reef islands, the smallest coastal deposits in the region, also show a variable trajectory of contemporary change including expansion, contraction and migration on reef surfaces (Kench and Mann, 2017; Sengupta et al., 2025).

Comparison of shoreline change rates must be undertaken cautiously as the rates are inherently dependent on the temporal and spatial scales over which they are calculated. Rates derived from millennial-scale processes, such as postglacial sea-level rise, reflect broad, long-term patterns that filter short-term variability. In contrast, short-term estimates over recent decades may be more greatly influenced by short-term variability. Neither set of observations adequately captures the episodic and often extreme impacts of singular events that can substantially modify coastal configuration. Consequently, interpretation of long-term rates of coastline change must also remain cognisant of the magnitude of variability introduced by short-term processes, which can strongly modulate or punctuate longer-term trends.

Existing geological and contemporary studies reveal broad coastal system-scale sensitivities to sea-level change that provide first-order approximations of thresholds of change in coastal trajectories. Paleo-reconstructions show that at rates of sea-level change greater than 10 mm/yr, and exceeding 15 mm/yr in the late Pleistocene to early Holocene (Figures 3, 5, and 6), coastal response across SEA was characterised by broad-scale inundation and rapid coastal regression, as the rate of sea-level change overwhelmed sediment delivery. In contrast, when rates of RSLR fell below 10 mm/yr in the mid- to late Holocene (and the relative importance of sediment delivery was greater than sea-level change), there was active coastal aggradation and progradation. Consequently, it is not surprising that the magnitude of rates of coastal change reported in contemporary studies is comparable to those of the mid-late Holocene, as the coastal systems are maintaining a morphodynamic equilibrium within the lower range of RSLR rates. Collectively, these studies suggest that an order of magnitude threshold exists at ~10 mm/yr SLR that may indicate a tipping point for coastal system behaviour in the region (Figure 5), as has been suggested elsewhere (Miselis and Lorenzo-Trueba, 2017; Wright and Thom, 2023). Specifically, rates of RSLR on the order of ~10 mm/yr may exceed the capacity of SEA coastal systems to maintain morphodynamic equilibrium, beyond which widespread inundation or large-scale reorganisation (regression) can be envisaged (Figures 5 and 6). Of note, sea-level projections across SEA over the next century indicate rates of SLR will vary regionally and range from 2.53–11.2 mm/yr (SSP1–2.6 scenario) to 6.6–29.1 mm/yr (SSP5–8.5) (Ng et al., 2024). Such spatial variation indicates that while many coastal systems are currently (and may remain) beneath the 10 mm/yr threshold and can maintain their morphodynamic equilibrium, other coastal sites are approaching, or have already exceeded this threshold. It is important to stress that each specific coastal system is likely to have a specific threshold value, and this will vary regionally depending not only on the rate of RSLR but also on sediment supply. Anthropogenic impacts on sediment supply are one significant difference that may alter such thresholds (positive and negative) from those observed in the Holocene.

Further resolving the magnitude, rate and direction of coastal change and specific sea-level thresholds is fundamental for future management and adaptation planning, particularly considering the threats of global climate change and SLR, subsidence and anthropogenic pressure. Driven by this concern, there has been an increase in studies that model and predict changes in coastal systems, yet such approaches remain fragmented across disciplinary and spatial scales. Modelling efforts can be grouped into those that (i) adopt process-based models to simulate hydrodynamics and sediment fluxes in specific coastal systems such as deltas (An et al., 2025; Van Binh et al., 2022) or coastal cells (Tran et al., 2022; Jefri et al., 2026); (ii) apply morphodynamic models, at local scales, to assess the impact of extreme events on coastlines or impacts of anthropogenic activities; (iii) combine process-based and probabilistic models to simulate flood hazards under future sea-level rise scenarios and (iv) adopt remote sensing and spatial analytics to resolve past coastline trends and project future trajectories. However, each of these approaches has limitations with respect to the temporal scale of prediction, spatial application (from local to regional) and validation based on ground-truthed data, and although process-based models are becoming increasingly sophisticated, advances in explicit modelling of geomorphic change remain challenging. Key challenges to developing both local and regional specific models are the inclusion of key drivers of coastal change in the region, beyond sea level, that include vertical land movement, particularly subsidence in major deltas and requires coupled hydrogeological and InSAR-based frameworks; improved resolution of sediment supply at local to regional scales; inclusion of ecosystem-based modelling, especially of biogeomorphic processes and the feedbacks on shoreline morphodynamics; incorporation of anthropogenic intervention and an explicit focus on simulating and predicting coastal morphological adjustments.

In addition, effective planning is also constrained by insufficient data, at the necessary scale, and across the diversity of coastal landform types in SEA. First, the geographic spread of studies of paleo- and contemporary coastal change is limited. Paleo reconstructions of coastal evolution in response to past sea-level change provide instructive insights into the styles, rates and spatial dimensions of coastal response, particularly where coasts retain their natural function. However, further studies that include all coastal types are necessary to better resolve the coastal response to boundary controls and constrain the development of modelling approaches.

Studies of contemporary coastal change, coastal hazards and management also highlight significant regional differences in the number of studies and coastal landform types examined (see Dong et al., 2024). For example, greatest effort has occurred in Vietnam where studies have assessed shoreline dynamics of the major delta systems (e.g. Anthony et al., 2015; Ve et al., 2021) as well as the central coast (e.g., Quang Tuan et al., 2017; Liem et al., 2020), with Lappe et al. (2022) undertaking an assessment of the entire coast. Elsewhere, studies have been fewer in number, lack national coverage and in some countries no systematic analysis has been undertaken (Table 1). Consequently, apart from Vietnam, studies are not representative of coastal landform types within countries. In lieu of comprehensive site-specific studies, regional assessments of coastal change in mainland SEA (Song et al., 2020) and islands (Zhang et al., 2021) provide valuable approximations of coastal change. The regions’ coasts are also included in global assessments of shoreline change (Luijendijk et al., 2018; Vousdoukas et al., 2020). However, there remain large uncertainties in these aggregate analyses at the local scale and there is an urgent need for comprehensive site-specific studies to validate results and yield higher resolution datasets.

Second, many existing studies focus on sites where specific hazard impacts are expressed. For example, multiple studies have examined the magnitude and impacts of anthropogenically forced subsidence on flood hazards on the north coast of Java (Bott et al., 2021), Semarang (Abidin et al., 2013) and Ho Chi Minh city (Tay et al., 2022). Furthermore, numerous studies have examined erosional hotspots such as in the northern Gulf of Thailand (Bidorn et al., 2021; Sok et al., 2022) or Terengganu on the east coast of peninsula Malaysia (Bagheri et al., 2019; Rojahan et al., 2022). While such studies are important due to pressing management and vulnerability issues, caution must be exercised in extrapolating such results to characterise coastal change across the wider region. In particular, the data show that erosion and inundation are not the sole coastal trends, and while these are apparent at specific localities, coastal stability, progradation and uplift are actively occurring elsewhere. Resolving this patchwork of responses in higher resolution is paramount to support robust local and national-scale adaption planning.

Third, the attribution of contemporary coastal change is generally poorly constrained across the region. Most reliable attributional insights have emerged from sites where a key driver is clearly resolved, or locations which have been impacted by extreme events. For example, the role of anthropogenically forced subsidence is well-documented at specific localities (Nicholls, 2021). In addition, tectonic processes such as the 2004 Sumatra-Andaman earthquake caused several meters of coseismic uplift and subsidence in parts of northern Sumatra (Meltzner et al., 2006) and triggered post-seismic viscoelastic deformation at distal locations (Peng et al., 2024). These processes promote rapid and detectable changes in the coast. The immediate and long-term impacts of tsunamis and typhoons on coastal landforms and coastal hazards have also been examined (Liew et al., 2010; Ghadamode et al., 2024). However, beyond these clear examples, many studies have been unable to determine specific drivers of change due to several reasons. First, the datasets of coastal change are typically of insufficient length and at different temporal resolution to datasets that characterise the primary drivers of change (e.g., sea level, tectonics), preventing robust statistical exploration. Second, the detectable effects of long-term changes in RSLR are likely masked by short-term variability in the coastal processes. Third, sea level and climatic change are often uncritically invoked as drivers of change. While accelerated sea-level rise is a pressing issue in the region, patterns of RSLR are variable (Figure 2) and in many areas geological and anthropogenic processes may exert a more dominant influence. Differentiating coastal sites where RSLR is likely to exert a major influence from those where it has less influence is paramount. Fourth, while the anthropogenic footprint on the coast of SEA is expanding (Song et al., 2020; Zhang et al., 2021), attribution must resolve direct and indirect impacts. For example, detection of direct shoreline modification is readily quantifiable; however, indirect effects due to alongshore alterations in the sediment budget or process regime are difficult to resolve.

This review underscores that coastal change across SEA is complex and, rather than a uniform trajectory of erosion and inundation, the region exhibits a mosaic of responses, including stability to rapid progradation and migration of islands, driven by diverse geological, climatic and anthropogenic processes. Accurate characterisation of these dynamics and attribution of coastal change across SEA remains fundamentally constrained by sparse spatial coverage of studies, uneven representation of the region’s diverse coastal landform types and the limited temporal resolution of available datasets. Existing observations are often geographically clustered, disproportionately focused on high-risk or rapidly changing sites, and derived from records that are too short or discontinuous to robustly resolve the relative influence of key drivers operating across multiple timescales. As a result, distinguishing the roles of sea-level rise, sediment supply, vertical land motion, ecological processes and anthropogenic modification remains challenging, limiting confidence in both empirical interpretations and model calibration. Addressing these constraints requires a step change in future empirical research design. Priority should be given to establishing sustained, spatially distributed monitoring networks that resolve both morphological change and sediment budgets, including continuous sediment fluxes, across coastal systems, alongside improved chronological control through high-resolution dating frameworks that link paleo and contemporary records. Equally critical is the integration of geomorphic and ecological datasets to better resolve biogeomorphic feedbacks, particularly in mangrove and reef-associated coasts. Such coordinated, multi-scalar datasets will provide the robust empirical foundation necessary to parameterise, validate and refine next-generation coastal models across the region to support robust adaptation.

## Data Availability

Data availability is not applicable to this article as no new data were created or analysed in this study.

## Long descriptions

### Table 1. Long description

Summary of coastal attributes, and dominant coastal landform types of countries in southeast Asia.

Navigate back to Table 1..
