# Supplementary material for: Coastal change in Southeast Asia from geological to contemporary timescales: a review
Source: Camb Prism Coast Futur. 2026 Jun 16;4:e16. doi: 10.1017/cft.2026.10039 (PMC13370299; doi:10.1017/cft.2026.10039)
Supplement: Peer review history

# Reviewer report: Review: Coastal change in Southeast Asia from geological to contemporary timescales: a review — R0/PR2

**DOI:** 10.1017/cft.2026.10039.pr2

**Authors:** Colin D. Woodroffe

**Affiliations:** School of Science, University of Wollongong, Australia

This is a useful review that summarises the geological and contemporary patterns of coastal change for one of the most densely populated regions on Earth. It outlines the complexity of modern shoreline trends, emphasising variability in terms of tectonic setting and providing a summary of observed progradation of some coasts and retreat on others, noting that extensive areas appear to have remained stable over recent decades. It notes that rates of relative sea-level rise are varied across the region and that there are natural geomorphological and anthropogenic reasons for the variability in coastal behaviour. It makes a comparison of these ongoing changes with the rates at which sea level, and consequently the shoreline, changed during the postglacial, the past 20,000 years. Unexpectedly, it implies that modern rates of change are in many cases of similar magnitude to those that occurred as the Sunda Shelf was flooded.

The paper is accompanied by some excellent diagrams.

The comparison of geological and contemporary timescales (as phrased in line 404) is a central feature of this review, and this could be made clearer from the start, using these terms earlier in the paper (instead of ‘paleo and modern’ in line 50 for example).

The title could be clearer. ‘Transforming coasts’ does not convey this essence of past and contemporary timescales; indeed, it could be interpreted in a social science context to indicate the ongoing human transformations of the coast, given the very significant anthropogenic alterations to many of the heavily populated shorelines.

It is surprising that so little is said about those human impacts. The emphasis of the paper is on natural change. There is brief reference in line 21, but it is not until line 471 that the significant role of human impacts is addressed and then divided into direct and indirect effects.

A constraint on comparison of averaged rates of change is the time period over which rates are calculated. The spatial and temporal variability of shoreline change is complex, as this paper emphasises. The rate at which a shoreline transgressed the landscape during rapid phases of postglacial sea-level rise determined at large spatial scale and millennial timescale will provide a different average from the short term rate derived from comparison of satellite imagery. Neither capture the rapid rate that may occur locally in response to a single extreme extent. The 2004 tsunami, for example, resulted in devastation of many shorelines, but its impact is more obscure when included in rates of change observed over decades. Line 46, in the Abstract, ‘contemporary rates of change rival those of the Holocene’ is a bold statement which deserves greater clarification (perhaps ‘rival’ is not the most appropriate word). Line 344 indicates that the changes were ‘equally substantive’, it is not clear what that means (particularly as following sentences indicate 70% has remained stable; see also lines 418-420).

There are 3 or 4 mentions of modelling, most of which use the same phrasing ‘modelling framework’. It would be useful to clarify what is meant here; the word framework seems unnecessary.

Line 63 – 9 nations – there are 10 listed in Table 1.

Line 147 – More could be said about subsidence of deltas.

Line 240 Section 3. This long paragraph contains, ‘first, second, third, fourth’ but it is not clear how these are connected; they do not seem to be successive time periods. Consider phrasing this differently. If they are time periods, their timing is unclear as sometimes the numbers are X,000 years ago, whereas elsewhere it is the duration of the period. Several edits are needed (line 247 ‘nature experienced’ .. line 249).

Line 279 – edit

Line 322 and 336 – edit

Line 342 – reword ‘excursions’

Line 391 – sentence without verb

Line 415-6 – This sentence seems out of place, and it is not clear what it means

Line 456 – is ‘exaggerated’ the best term?

The global shoreline analysis papers of Luijendijk and Vousdoukas are cited twice, but on neither occasion are any details given.

Caption to figure 2 has three sentences starting ‘circles are’; it could be clearer.

# Reviewer report: Review: Coastal change in Southeast Asia from geological to contemporary timescales: a review — R0/PR3

**DOI:** 10.1017/cft.2026.10039.pr3

**Authors:** Jorge Lorenzo-Trueba

This manuscript provides a synthesis of coastal landform change across SEA. By integrating paleo-reconstructions with contemporary remote sensing observations, the author highlights the dynamic and non-uniform nature of coastal change in the region. The discussion of spatial variability in RSLR, particularly as driven by tectonic VLM, is clear.

The review challenges simplified narratives of uniform coastal erosion under RSLR and shows that coastal response across SEA reflect a complex interplay of geological, climatic, ecological, and anthropogenic drivers. This synthesis is particularly important given the region’s high population density, rapid environmental change, and global significance as a coastal hotspot of sediment flux, ecosystem services, and climate vulnerability. I provide some suggestions aimed at improving the quality of the manuscript below.

The manuscript presents a synthesis of geological evolution and contemporary shoreline change, but the conceptual framework linking boundary conditions to coastal morphodynamic response could be more clear. While key drivers such as tectonics, RSLR, climate forcing, sediment supply, and anthropogenic impacts are discussed, they are largely presented sequentially rather than integrated. The manuscript would benefit from a schematic or conceptual model illustrating how these drivers interact to produce different coastal trajectories across major coastal typologies (e.g., deltas, reef islands, mangrove coasts, strand plains). This would help clarify the mechanisms underlying the spatial mosaic of responses emphasized throughout the manuscript.

The manuscript calls for “integrated modeling frameworks” in both the abstract and conclusion, and notes that “no robust projections exist for how coasts will physically transform over the next century”. This is an important point in my opinion. However, there is limited discussion of the current state of methods used to attribute and predict coastal change in this region. The manuscript would benefit from a subsection that evaluates existing predictive approaches (e.g., sediment budget analyses, morphodynamic modeling, integration of remote sensing with in situ observations). It would be useful to clarify why standard modeling approaches have been insufficient in SEA to date. For instance, how do current models struggle to incorporate the unique boundary conditions highlighted in this review, such as rapid tectonic VLM, biogeomorphic feedbacks (mangroves/reefs), and heavy anthropogenic modification? I think this would strengthen the paper’s conclusion without requiring an exhaustive literature review of all coastal models.

The manuscript highlights that contemporary rates of coastal change rival those of the Holocene. I agree this is an important point, but the comparison involves different temporal scales (millennial-scale geological reconstructions versus decadal remote sensing observations). It would be helpful to include a brief discussion acknowledging the limitations of comparing long-term averaged rates with short-term, potentially episodic observations, and clarifying how such comparisons should be interpreted.

Section 4 highlights significant rates of contemporary progradation, particularly in Vietnam and Myanmar. While intense human activity is mentioned as a contributing factor, the relative roles of natural sediment supply and anthropogenic modification could be more clearly distinguished. In many SEA coastal systems, human activities strongly influence coastal responses through upstream damming, sediment trapping, dredging, land reclamation, aquaculture clearing, and groundwater extraction. Because these activities have opposing geomorphic effects (e.g., subsidence and damming typically drive retreat, while reclamation artificially advances the shoreline), clarifying whether the observed ‘progradation’ reflects natural sediment dynamics or direct artificial land-building would significantly strengthen the interpretation of these contemporary trends.

The author identifies that attribution of coastal change is constrained by sparse spatial coverage, uneven representation of landform types, and limited temporal resolution of data. The author could also briefly discuss how future empirical research in SEA could be designed to better support modeling efforts (e.g., continuous sediment flux measurements, improved chronological control, integration of geomorphic and ecological datasets).

Minor comments:

Line 67: “beach ridge pains” → “plains”

Line 118: “multipe micro-plates” → “multiple micro-plates”

# Reviewer report: Review: Coastal change in Southeast Asia from geological to contemporary timescales: a review — R0/PR4

**DOI:** 10.1017/cft.2026.10039.pr4

**Authors:** Hajime Kayanne

General Assessment

This manuscript provides a timely and valuable synthesis of coastal change across Southeast Asia, addressing a region of critical importance under accelerating sea-level rise and environmental change.

The review makes three particularly important contributions:

It comprehensively integrates tectonic processes, sea-level change, and climatic–oceanographic forcing, including their strong spatial variability across Southeast Asia.

It systematically summarizes coastal responses for major landform types (e.g., strand plains, deltas, and coral reef systems), highlighting differences in geomorphic behaviour.

It clearly demonstrates that coastal responses to sea-level rise are not uniform or static (i.e., simple inundation), but instead dynamic, including progradation, stability, erosion, and landform migration.

These contributions are significant and provide an important conceptual foundation for understanding coastal change in Southeast Asia.

At the same time, several aspects could be further strengthened. Some of the suggestions below would require substantial revision; however, even if full implementation is not feasible at this stage, I strongly encourage the author to acknowledge these points explicitly as key directions for future research.

Major Comments

1. Need for clearer linkage between “dynamic response” and prediction/adaptation

While the manuscript convincingly demonstrates that coastal systems respond dynamically to sea-level rise, it remains unclear how this conceptual understanding can be translated into prediction or adaptation frameworks.

In particular, the manuscript would benefit from clarifying:

How the balance among key controls (sediment supply, ecological condition such as coral reefs and mangroves, and human modifications) governs whether coasts undergo progradation, stability, or erosion/inundation.

Whether there are identifiable thresholds or boundary conditions that lead to submergence or rapid retreat.

At present, the discussion remains largely conceptual. Including schematic diagrams or representative case examples for each major landform type (e.g., delta, strand plain, coral reef coast) would greatly enhance clarity and applicability.

2. Expansion of Figure 5 toward threshold interpretation

Figure 5 provides an important synthesis of sea-level rise rates and coastal system responses, particularly for coral reefs and mangroves.

This figure could be further strengthened by:

Indicating the range of projected sea-level rise rates for the current century, and

Extending the conceptual framework to include other major landform types, such as deltas and coastal plains.

Such additions would allow the figure to better bridge paleo-observations and future coastal response.

Minor Comments

(1) Representation of Meltwater Pulse 1A (MWP-1A)

In Figure 5, the sea-level rise between 15–9 ka appears relatively smooth, whereas the rapid rise associated with MWP-1A is expected to stand out more prominently. Please confirm whether this feature is adequately represented and consistent with the cited data.

(2) Geographic clarity and mapping of coastal characteristics (Figure 1 and text)

The manuscript refers to many geographic locations that are not clearly indicated in Figure 1, which may limit accessibility for readers less familiar with Southeast Asia.

In addition, the figure could be further improved by:

Incorporating the distribution of major coastal geomorphic types (e.g., deltas, strand plains, coral reef coasts), which would help readers connect the regional discussion with spatial patterns.

If volcanic islands are shown, it may be more relevant in the context of this manuscript to explicitly plot coral reef distributions, which are central to the arguments on biogeomorphic coastal response.

Final Recommendation

This is a strong and well-structured review that makes an important contribution to coastal geomorphology and climate adaptation studies.

I recommend publication after revision. While some of the points raised above may require substantial additions, even partial incorporation or clear acknowledgment of these issues as future research priorities would significantly strengthen the manuscript.

# Editor report: Recommendation: Coastal change in Southeast Asia from geological to contemporary timescales: a review — R0/PR5

**DOI:** 10.1017/cft.2026.10039.pr5

**Affiliations:** Division of Earth and Climate Scinces, Nicholas School of the Environment, Duke University, United States

I agree with all the reviewers that this wide-ranging review of coastal change in SEA is a valuable contribution. Some moderate additional work would, as suggested by the reviewers, improve the paper and likely enhance its impact. 

All the reviewer comments seem potentially helpful in improving the paper. Here I highlight what seem like central points raised by the reviewers. 

Perhaps the most important additions, as pointed out by reviewers 2 and 3, would be some form of summary synthesis, or conceptual framework, for how the different drivers of coastal change combine in different ways in different places to produce the various end members of coastal change behaviors. As it is presently written, parts of the review seem like a list of behaviors exhibited, and lists of the drivers of coastline change. Adding more analysis connecting them would be welcome, perhaps in the form of a schematic or conceptual model (as suggested by Reviewers 2 and 3). 

Alternatively, or in addition, a discussion of what types of studies are needed to produce a better ability to synthesize conceptual or quantitative models to better understand and forecast the array of different coastline types and behaviors would be valuable (as suggested by Reviewer 2). 

When revising the paper, please ensure that acronyms are defined (e.g. line 342) and proofread for minor grammar improvements (e.g. lines 243-245, 256, 317-328, 430 and 474). 

# Editor report: Decision: Coastal change in Southeast Asia from geological to contemporary timescales: a review — R0/PR6

**DOI:** 10.1017/cft.2026.10039.pr6

**Affiliations:** Geography, Cambridge University, United Kingdom of Great Britain and Northern Ireland

No accompanying comment.

# Editor report: Recommendation: Coastal change in Southeast Asia from geological to contemporary timescales: a review — R1/PR8

**DOI:** 10.1017/cft.2026.10039.pr8

**Affiliations:** Division of Earth and Climate Scinces, Nicholas School of the Environment, Duke University, United States

I thank the Author for carefully addressing the reviewer comments, including by adding more synthesis and analysis (e.g. the conceptual model outlined in Figure 6) that strengthens the paper. I concur with the reviewers that this well written review constitutes a valuable contribution. I believe it is ready to be published in the present form.

Thank you.

# Editor report: Decision: Coastal change in Southeast Asia from geological to contemporary timescales: a review — R1/PR9

**DOI:** 10.1017/cft.2026.10039.pr9

**Affiliations:** Geography, Cambridge University, United Kingdom of Great Britain and Northern Ireland

No accompanying comment.
